# Extract of *Ginkgo biloba* exacerbates liver metastasis in a mouse colon cancer Xenograft model

**DOI:** 10.1186/s12906-017-2014-7

**Published:** 2017-12-02

**Authors:** Huan Wang, Xia Wu, Stephane Lezmi, Qian Li, William G. Helferich, Yueqing Xu, Hong Chen

**Affiliations:** 1 Department of Food Science and Human Nutrition, University of Illinois at Urbana-Champaign, Urbana, IL 61801 USA; 2 College of Food Science and Nutritional Engineering, China Agricultural University, Beijing, 100083 People’s Republic of China; 3 Department of Pathobiology, University of Illinois at Urbana-Champaign, Urbana, IL 61801 USA; 4 Medical College of Hebei University, Baoding, China; 5 Division of Nutritional Sciences, University of Illinois at Urbana-Champaign, 472 Bevier Hall, MC 182 905 S Goodwin Ave, Urbana, IL 61801 USA; 6 Current address: Huan Wang, Postdoctoral Scholar, Department of Human Genetics, University of California at Los Angeles, California, USA; 7 Current address: Qian Li, Senior Scientist, Mead Johnson Nutrition, Evansville, Indiana, 47721 USA

**Keywords:** EGb, Metastasis, Mapk, Proliferation, Gene expression

## Abstract

**Background:**

Metastasis refers to the spread of a primary tumor cell from the primary site to other locations in the body and it is generally associated with the severity of a tumor. Extract of *Ginkgo biloba* (EGb) contains various bioactive compounds and it exerts beneficial effects including improvements in brain function and reduced risk of cardiovascular diseases. On the other hand, increased risk of thyroid and liver cancers by EGb have been reported in animals.

**Methods:**

A colon cancer metastasis model was established using intrasplenic injection of a human colon cancer cell line, SW620-luc in athymic mice to investigate the potential impact of EGb on colon cancer progression. After tumor establishment, EGb was intraperitonically injected daily for 5 wks.

**Results:**

EGb significantly increased the rate of metastasis in mouse liver and decreased the number of necrotic and apoptotic cells in the metastatic liver when compared to the control. Meanwhile, EGb significantly induced proliferation of tumor cells in the metastatic liver, indicated by increased staining of Ki67 and H3S10p. mRNA expression of genes involved in cell cycle, metastasis, apoptosis, and oxidative stress were altered by EGb treatment in livers with tumors. Moreover, EGb activated the stress-responsive MAPK pathways in the liver with metastatic tumors.

**Conclusions:**

EGb exacerbated liver metastasis in a mouse colon cancer metastasis model. This is potentially due to the increased tumor cell proliferation involving stimulated MAPK pathways.

**Electronic supplementary material:**

The online version of this article (10.1186/s12906-017-2014-7) contains supplementary material, which is available to authorized users.

## Background

Colorectal cancer remains a major public health problem worldwide. It is the third most common cancer in men and the second in women worldwide (GLOBOCAN 2012). It is estimated that 694,000 people die from colorectal cancer and 1.2 million new cases are diagnosed each year (GLOBOCAN 2012). Furthermore, liver metastases occur in approximately 30% of all colorectal cancer patients and account for at least two thirds of deaths from colorectal cancer [1, 2].

*Ginkgo biloba* contains a rich mixture of bioactive compounds including flavonol glycosides, terpene trilactones, biflavones, alkylphenols, phenolic acids, proanthocyanidins, and polyprenols [3]. *Ginkgo biloba* seeds have been used in traditional Chinese medicine for centuries, and the leaf extracts of *Ginkgo biloba* (EGb) has been sold as phytochemicals in Europe and dietary supplements in U.S. since 1960s [4, 5]. However, recent studies using EGb have been controversial. For example, EGb is commonly used for its positive effects on improving memory and cognitive functions, reducing oxidative stress, and inhibiting proliferation in adenoid cystic carcinoma [6, 7]. Also, EGb has been widely used for treatment of cardiovascular and nervous system diseases such as peripheral and cerebrovascular disorders, ischemic and Alzheimer-type dementia [6, 8, 9]. This has been attributed to EGb’s ability to inhibit platelet aggregation and to prevent the formation of arteriosclerotic plaques [10]. Furthermore, it has been reported that EGb has anticancer effects in breast and gallbladder cancers [11, 12]. These effects potentially involve diverse mechanisms, including the reduction in the number of apoptotic cells [13–16], the maintenance of mitochondrial integrity [17–20], inhibition of cyt c release from mitochondria, induction of gene expression of the anti-apoptotic protein Bcl-2 [21–25], reduction in the gene expression of caspases [21, 24, 26–28] and DNA fragmentation [21, 24]. In addition, EGb up-regulates the mRNA expression of antioxidant enzymes such as mitochondrial SOD, GPx, GCLC, and HO-1 to decrease oxidative tissue damage [29–31].

On the other hand, data from a clinical trial showed that EGb intake is associated with increased risks of breast and colon cancers [32]. Furthermore, a recent report from the National Toxicology Program [33] shows that *Ginkgo biloba* increases the risk of thyroid cancer in F344/N rats and B6C3F1/N male mice as well as liver cancer in B6C3F1/N mice. However, the mechanisms behind the increased cancer risk by EGb are not well investigated or understood. Therefore, the main objectives of the current study were to investigate the effects of EGb on tumor development in the liver of colon cancer xenograft mice and to further asses potential underlying mechanisms related to angiogenesis, oxidative stress, and cellular proliferation.

## Methods

### Engineering colon cancer cells for bioluminescent imaging (BLI)

Firefly luciferase was used to illuminate colon cancer cells used in the non-invasive bioluminescent imaging. The luciferase gene was excised from the pGL3 luciferase reporter vector (www.promega.com) and was inserted into the pLPCX vector that contains the LTR from Moloney murine leukemia virus. The luciferase-pLPCX plasmid was then transfected into a viral packaging cell line 293 T–e for packaging retroviral particles and ecotropic delivery using SuperFect transfection reagent (Qiagen.com). Packaging cell line 293 T was kindly provided by Dr. Michael Kilberg at the University of Florida, Gainesville. SW620 cells (www.atcc.org; catalog # CCL-227) were infected with the luciferase-expressing retrovirus and the resulting SW620-luc cell line was established for stably expressing luciferase enzyme for in vivo luminescent imaging. Cell line verification was performed in the laboratory according to the guidelines by ATCC (Technical Bulletin No. 8, www.atcc.org). Cell line authentication has been performed according to the guidelines set by UKCCCR [34] and the latest authentication tests were performed by a commercial testing laboratory (Radil lab, Columbia MO).

### Extraction and analysis of EGb

Extract of *Ginkgo biloba* (EGb) used in the study was provided by Hebei Medical College (Hebei, China) using a procedure by Baweisong and associates (Chinese patent #00129807.0). Briefly, the crude extract of *Ginkgo biloba* (100 g) was dissolved in 666 mL methanol and sonicated for 15 min, followed by the addition of 1200 mL Ethyl and another sonication of 30 min. The mixture was ultra-filtered and dried using vacuum pump at 75 °C for 24 h. The purified product was dissolved in boiling ethanol water (17% *w*/w in water), decolorized with active carbon for 5 min, and filtered. The purified product was mixed with Na_2_S_2_O_5_ solution and 2 g of Ginkgolide B was added to the mixture and dissolved under heat. The mixture was then concentrated by filtration. EGb concentrate was adjusted to pH 7 by NaHCO_3_ before autoclave sterilization. Final product was packaged into 17.5 mg per ampule vial. Characterization of the purified EGb was conducted using HPLC. HPLC-grade acetonitrile was obtained from Fisher Scientific (Fisher Scientific, USA). The reference crude extract of *Ginkgo biloba* leaves (Lot No. 1160–200,001) and rutin reference standard were purchased from the National Institute for the Control of Pharmaceutical and Biological Products (Beijing, China). Ginaton was purchased from Dr. Willmar Schwabe Pharmaceuticals (Karlsruhe, Germany). All other chemicals were analytical grade. The rutin reference standard solution was accurately weighed and dissolved in methanol then diluted to appropriate concentration ranges for the establishment of calibration curves. Two g of reference of *Ginkgo biloba* was dissolved in 50 mL ethanol, and ultrasonic extracted for 30 min. After the addition of 50 mL ethanol, the extract was filtered through a 0.45-μm PTFE filter membrane, as EGb reference sample solution. Analysis was done using a Diamonsil C18 column (5-μm particle size, 250 × 4.6 mm i.d.). Separation was carried out with gradient elution procedure. Linear ratio of mobile phase A (acetonitrile) and B (1% H3PO4) was set as follows: 0~30 min, 15~17% A; 30~37 min, 17~16% A; 37~45 min, 16~20% A; 45~60 min, 20~25% A; 60~80 min, 25~15% A. The total run time was 80 min at a flow rate of 1 mL/min. The eluent was monitored by a diode array detector, and the detection wavelength was set at 360 nm. The injection volume of sample was 20 μL, and the column temperature was 30 °C.

### Animals and treatment

Twenty-four 4-wk.-old athymic mice were purchased from Charles River Laboratories (Charles River Laboratories, Wilmington, MA) and fed a modified AIN-76 diet (product #D12010101, Research Diets, Inc., New Brunswick, NJ). Animals were housed individually in standard polycarbonate cages in a humidity- and temperature- controlled environment on a 12-h light-dark cycle. After 2-wk. adaptation, animals were intra-splenically injected with 2.5 × 10^6^ of SW620-luc colon cancer cells. Tumor development and metastasis were monitored weekly by BLI of live animals. Briefly, BLI was conducted using a custom-made imaging system for live animals (Stanford Photonics, Palo Alto, CA), which includes a photon-tight dark box, bright field illumination, warming plate and a dual micro-channel plate ICCD camera (Mega 10-Z with cathode cooling). D-luciferin was freshly dissolved in PBS as a 150 mg/mL solution before administration. Injecting volume (μL) of D-luciferin was calculated as 10× body weight (g) and D-luciferin was injected intraperitoneally into each animal. Three min after the administration of D-luciferin, each mouse was anesthetized using isoflurane/oxygen gas from a vaporizer and placed face-up on a height-adjustable tray in the imaging system for whole body imaging. General anesthesia was maintained throughout the imaging. Photon emission was collected for a 3-min period using imaging software Piper Control (Stanford Photonics, Palo Alto, CA). At 10 wks post cancer cell injection, BLI imaging was used to determine tumor development and to assign animals into Tumor (animals with tumor development) and Normal (animals without detectable tumors) for subsequent EGb treatment.

**Fig. 1:**
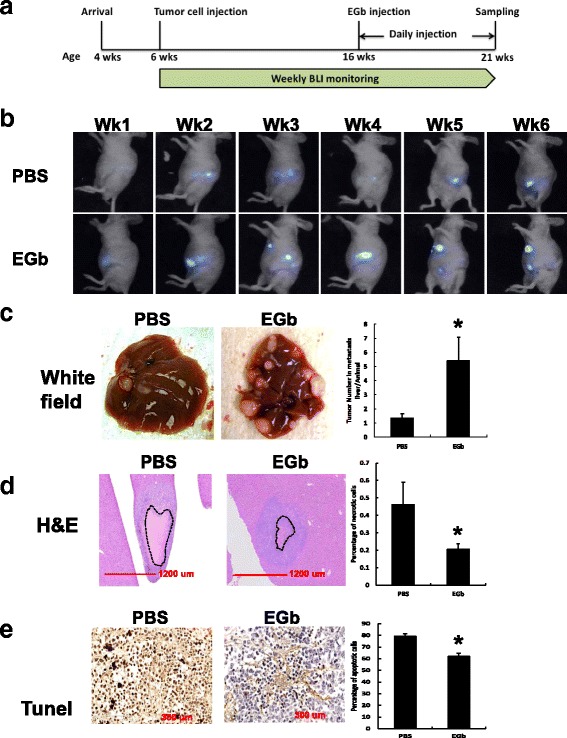
Characterization of EGb treated mice underwent intrasplenic injection of colon cancer cells. **a** Timeline of animal trial and design. Mice were received at 4 wks of age. Food intake and body weight measurement were performed weekly from 4-wk. to 21-wk. of age. At 6 wks of age, mice were injected with SW620-luc cells. Daily injection of EGb (PBS was used as the negative control) was conducted from 16 wks of age to 21 wks of age. Mice were sacrificed after 5 wks of EGb injection. **b** Bioluminescence Imaging (BLI). Mice were imaged weekly from wk. 1 to wk. 6 of intraperitoneal injection of PBS or EGb. Representative imaging was shown for mice with positive bioluminescent signals. **c** Characterization of mouse liver containing metastatic tumors in PBS and EGb mice. Representative pictures with liver tumors are in the left panel. Quantification of numbers of tumors in liver after EGb treatment is shown in the right panel. Y-axis represents the number of tumors in the metastatic liver per animal. **d** Necropsy and pathology by structural staining using H&E of mouse liver. Representative images with metastatic tumor in the liver were shown for PBS and EGb groups (5× magnification). Gross structure was stained in pink and nuclei were stained in purple. Dotted lines were used to circle the necrotic area in the tumors that were quantified. Right panel shows the quantification of necrotic area (%) in PBS and EGb treated mouse liver. Y-axis indicates the percentage of necrotic area within the tumor. **e** TUNEL analysis of apoptosis in liver tumors. Representative pictures are shown. Brown dots are the positively stained nuclei of apoptotic cells. Quantification of apoptotic cells in PBS and EGb treated livers are in the right panel. Y-axis indicates the percentage of apoptotic cells in the liver tumor. Asterisks (*) indicate statistical significance (*n* > 4 animals, *p* < 0.05) when compared to the PBS group

A total of 12 animals in the Tumor group and 12 in the Normal group were randomized into PBS (*n* = 6) and EGb (n = 6), generating 4 groups of animals with Tumor/PBS, Tumor/EGb, Normal/PBS, and Normal/EGb treatments. Normal groups of animals were included for later assessment of the potential effects of EGb in normal liver without tumor development. Animals in the EGb groups were intraperitoneally injected with EGb solution daily for 5 wks with a dose of 35 mg/kg body weight (3.5 mg/mL EGb diluted in PBS). The dose of EGb was based on a previous publication by Rojas et al. [35]. Five wks later, all animals were euthanized by CO_2_ asphyxiation before being decapitated for sample collection. A part of liver, spleen and other tissues were fixed in formalin, while the remainder of the liver was snap frozen in liquid nitrogen and stored at −80 °C for further analyses. The experimental design is outlined in Fig. 1a. All experiments were conducted in accordance to the National Institutes of Health Guide for the Care and Use of Laboratory Animals and were approved by the University of Illinois Institutional Animal Care and Use Committee.

### Histological observations

Animals were euthanized by CO_2_ asphyxiation before being decapitated for sample collection. Liver tissues were fixed in 10% buffered formalin for 24 h, then trimmed and embedded in paraffin. Four-μm-thick sections were stained with hematoxylin and eosin (H&E) and tumor number and size (in diameter) were analyzed by obtaining images using a NanoZoomer Slide Scanner and NDP View software (Hamamatsu, Bridgewater, NJ). Ten 20× light microscopic fields were examined on each section for each liver. Six representative sections from different locations in each liver were analyzed. The presence of micro-metastases at necropsy was evaluated by the veterinary pathologist Dr. Stephane Lezmi.

### Quantitative real-time RT-PCR

Total RNA was isolated from mouse liver using Tri-reagent (Sigma-Aldrich, St. Louis, MO) according to the manufacturer’s instructions. RNA concentration was measured by Bio-Rad spectrophotometry (Bio-Rad laboratories Inc., Irvine, CA). cDNA was synthesized from 2 μg total RNA using High Capacity cDNA Reverse transcription Kit (Applied Biosysterms, Foster City, CA) in a DNA 2720 Thermal Cycler (Applied Biosystems, Foster City, CA). The program was as follows: 25 °C for 10 min, 37 °C for 120 min, and 85 °C for 5 s. Twenty-five ng of cDNA was analyzed using quantitative real-time PCR and relative mRNA level was detected using the SYBR Green fast master mix (Quanta BioSciences, www.vwr.com) with a 7300 real-time PCR system (Applied Biosystems, Foster City, CA). The reaction was as follows: 40 cycles of 95 °C for 15 min, 95 °C for 15 s and 60 °C for 60 s. After amplification, dissociation curves were acquired by stepwise increases from 55 °C to 95 °C to ensure a specific product amplified in the reaction. Standard curves with slope of −3.30 ± 0.20 and R^2^ ≥ 0.95 were accepted. The following housekeeping genes whose expression were not affected by the treatment were used to calculate the geometric mean to normalize raw PCR values: *L7a, GAPDH, Tbp, Actb, 36B4* and *HPRT1*. The equation for calculation of geometric mean is ${\left(\prod \limits_{i=1}^n{a}_i\right)}^{1/n}=\sqrt[n]{a_1{a}_2\cdots {a}_n}$where a_1_-a_n_ are PCR results for individual housekeeping genes. Primers were designed using Vector NT I software (Invitrogen Corporation, Carlsbad, CA) and synthesized by Integrated DNA Technologies (www.idtdna.com). Primer sequences used in the experiment are listed in Additional file 1: Table S1.

### Western blotting

Frozen liver (200 mg) was ground in liquid nitrogen and suspended in 500 μl of protein lysis buffer (0.125 mol/L Tris–HCl, pH 6.8, 1% SDS, 0.04% bromophenol blue, and 20% glycerol, *v*/v) with 1× proteinase inhibitor (Roche applied sciences, Indianapolis, IN) and phosphatase inhibitor cocktail 1 and 2 (Sigma-Aldrich, St. Lois, MO). Samples were then sonicated with 25 pulses at power setting 2 (Fisher Scientific model 100 Sonic Dismembrator, Pittsburgh, PA). Twenty μg of diluted protein were size-fractionated on a 12% Tris–HCl polyacrylamide gel and transferred to a PVDF membrane (Bio-Rad, Hercules, CA). The membrane was then incubated with a blocking solution [Tris-buffered saline/Tween (TBS/T), 20 mmol/L Tris–HCl, pH 7.6, 137 mmol/L NaCl, and 0.1% v/v Tween-20] containing optimal amount of bovine serum albumin (BSA) or nonfat dry milk (NFDM) for 1 h at room temperature. Primary antibodies were diluted in the blocking solution according to the manufacturers’ instructions and incubated with the membrane for 3 h at room temperature. Antibodies used are listed in the Additional file 1: Table S2. Primary antibody solutions were prepared as follows: phospho-p38 MAPK (1:5000 in 5% BSA), p38 MAPK (1:1000 in 10% NFDM), phospho-p44/42 and p44/42 (1:1000 in 10% NFDM), and phospho-JNK and JNK (1:1000 in 5% BSA). Subsequently, the membranes were washed with TBS/T for 5 X 5 min. HRP-conjugated secondary antibody was diluted to 1:10,000 in a blocking solution containing 1% (*w*/*v*) NFDM and incubated for 1 h at room temperature. After 5 times of 5-min wash with TBS/T, membranes were incubated with enhanced chemiluminescence reagent SuperSignal West Dura (Pierce; Rockford, IL). Signal was detected and quantified using ChemiDoc XRS imaging system (Bio-Rad).

### Immunohistochemistry (IHC)

A standard three-step streptavidin–biotin complex immunohistochemical assay was performed on paraffin sections for the detection of Ki-67 and phosphor-Histone H3 (Ser 10). Sections were deparaffinized and rehydrated in gradient concentrations of xylene and ethanol and then in distilled water. Sections were treated with 3% H_2_O_2_ to quench endogenous peroxidase activity. Antigen retrieval was performed by microwave pretreatment in 0.01 M citrate buffer for 25 min. After cooling, non-specific binding of the antibody to sections was blocked with 10% rabbit serum, Ki-67 antibody (BD#550609, Pharmingen, San Francisco, CA, USA) was applied in a 1:3000 dilution and incubated at 24 °C for 2 h. For pH3S10, antibody was diluted to 1:500. A subsequent incubation of 30 mins with biotinylated anti-rabbit serum was followed by a 30-min incubation using the Vectastain Elite ABC anti-rabbit kit (Vector Laboratories). The peroxidase was then developed in DAB (Diaminobenzidine, Amresco Inc. OH, USA) with H_2_O_2_. The sections were counterstained with hemotoxylin, dehydrated in gradient ethanol solution, cleared in xylene and mounted. One known liver tumor sample was included for positive control and for negative control when the primary antibody was replaced with 10% rabbit serum (manufacturer’s instructions for Vectastain Elite ABC anti-rabbit kit).

### TUNEL assay

Early cell apoptosis was detected using the ApopTag TM peroxidase kit (Oncor, Gaithersburg, MD) per the manufacturer’s instructions. Briefly, sections were first deparaffinized and rehydrated. The cell membranes were then digested with 20 μg/mL of proteinase K. The endogenous peroxidase activity was quenched with 3.0% hydrogen peroxide and sections were incubated with the equilibration buffer containing dUTP-digoxigenin. TdT enzyme was added and the reaction was stopped by dipping the slides in the stopping buffer. Then an anti-digoxigenin antibody conjugated with peroxidase was added, followed by 0.05% of the substrate, 3′ 3′-diaminobenzidine tetrahydrochloride (DAB). One sample without the addition of reaction mixture was used as a negative control and the same sample using DNase I to induce DNA strand breaks was used as a positive control according to the manufacturer’s instructions for ApopTag®Peroxodase In Situ Apoptosis Detection Kit.

### Cell cycle analysis

Fifty mg of frozen liver was ground by a mortar and pestle in liquid nitrogen and homogenized in PBS with 5% FBS. The homogenized samples were centrifuged twice at 1.12 × 1000 *g* for 2 min to collect the supernatant and the resulting cell suspension was washed twice with PBS containing 5% FBS. Cells were counted using a hemocytometer and the concentration was adjusted to ~1 × 10^6^ cells/mL. The cells were pelleted by centrifugation, resuspended in PBS, fixed, and stored at −20 °C until future analysis. Propidium iodide was used to stain DNA and cell cycle analysis was performed using a BD Biosciences LSR II flow cytometer at the Flow Cytometry Facility (The W.M. Keck Center for Comparative and Functional Genomics, University of Illinois at Urbana-Champaign).

### Statistical analysis

Fisher’s exact test was used to compare numbers of implantation and liver metastases between EGb group and PBS group (Table 1). Student *t*-tests were used to compare EGb and PBS groups for all remaining analyses. Each data point represents the mean ± SEM. Differences were considered significant at *p* < 0.05. The geometric mean was used to normalize data from RT-PCR for relative mRNA expression.

**Table 1:** Necropsy data

	Implantation rate^a^	Liver metastasis rate	Tumor size (mm)	
			Spleen	Liver and others
PBS	58%	38%	8.7 ± 1.8	10.4 ± 4.1
EGb	71%	50%	17.3 ± 3.6^b^	7.4 ± 2.0

^a^Implantation rate: Percentage of animals developed cancer after splenic injection; liver metastasis rate: Percentage of animals with tumors that developed liver tumors

^b^statistically significant when comparing to PBS control (*n* > 4, *p* < 0.05)

## Results

### EGb exacerbated liver metastasis in mice

In order to determine whether EGb affects tumor development in mice, athymic mice were used to establish a human colon cancer xenograft model. Human colorectal cancer cells, SW620-luc cells were injected into the spleen to induce tumor development before EGb or PBS control was injected 10 weeks later. Tumor growth in PBS- and EGb-treated mice was monitored weekly by BLI (Fig. 1b). Live monitoring of luciferase activity and location of SW620-luc tumor cells showed that bioluminescent signals increased in both PBS- and EGb-treated mice over time (Fig. 1b). Moreover, the EGb group developed tumor metastasis from spleen to an upper portion of the mouse body over time (Fig. 1b). Necropsy at the end of the study showed that the implantation rate (the percentage of mice that developed tumors) in PBS and EGb groups was 58% and 71%, respectively (Table 1). Additionally, liver metastasis rate (the percentage of mice that developed liver tumors among mice with tumor implantations) was 38% and 50%, for PBS and EGb groups respectively (Table 1). EGb significantly increased the total number of tumors in the liver compared to PBS (Fig. 1c, *p* < 0.05). Furthermore, size of spleen tumors was significantly larger in EGb mice compared to PBS (Table 1). Tumor sizes from all other tissues and organs were not statistically different between EGb and PBS groups (Table 1).

The gross structure of the liver with metastasis – including necrotic cells, mitotic cells and regular hepatocytes – was histologically examined using H&E stained liver sections (Fig. 1d). EGb mice had significantly smaller necrotic areas in the liver when compared to the PBS group (Fig. 1d, *p* < 0.05). Furthermore, early apoptosis analysis using the TUNEL assay confirmed that EGb significantly decreased apoptosis in the metastatic liver when compared to the PBS group (Fig. 1e, *p* < 0.05). In summary, EGb treatment significantly increased metastasis to liver from the spleen, and decreased necrosis and apoptosis in tumor when compared to the PBS control group in mouse liver.

### EGb increased mitosis and proliferation in the mouse liver

**Fig. 2:**
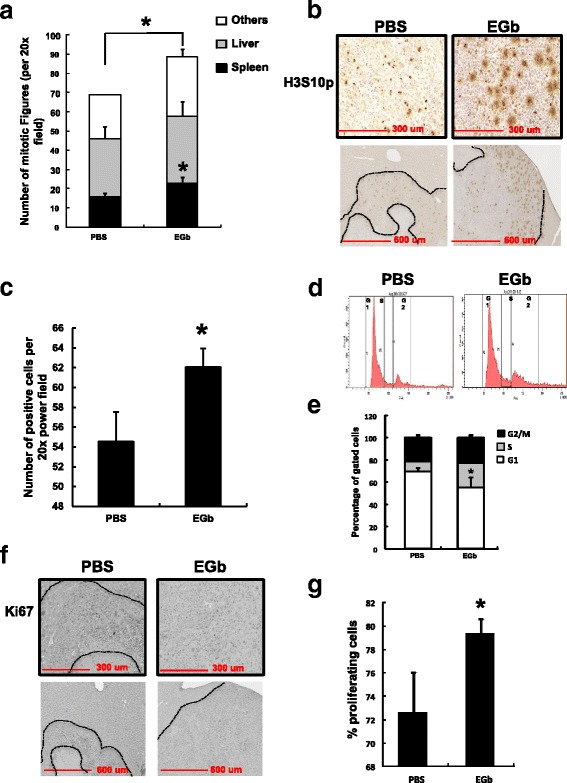
Mitosis and cell cycle in metastatic tissues. **a** Mitosis in the spleen, liver and other sites in mice with metastatic tumors. Black bars: numbers of mitotic figures in spleen, gray bars: numbers of mitotic figures in liver, open bars: numbers of mitotic figures in other abdominal tissues including skin and smooth muscle, adipose and mesentery lymph node. Y-axis is the number of mitotic figures per 20× power field. **b** Immunohistochemical staining of phosphorylated histone H3 serine 10 (H3S10p) in the metastatic liver. The upper panels show 20× power field and the lower panels show 10× power field of cross-sectioned mouse liver. The dotted lines outline tumor area without necrosis. **c** Quantification of H3S10p staining in the metastatic liver. Y-axis represents the number of positive cells per 20X power field. **d** Cell cycle analysis in mouse liver with metastasis. X-axis represents the signal intensity by PI staining and Y-axis indicates the total cells count. **e** Quantification of cell cycle analysis. Y-axis represents the percentage of gated cells (%). Open bars: % of cells in G0/G1 phase, grey bars: % of cells in the S phase, black bars: % of cells in the G2/M phase. **f** Immunohistochemical staining of Ki67 in the metastatic liver. The upper panels show 20× power field and the lower panels show 10× power field of cross-sectioned mouse liver. The dotted lines outline tumor area without necrosis. **g** Quantification of Ki67 staining in the metastatic liver. Y-axis represents the percentage of proliferating cells (%). Asterisks (*) indicate statistical significance (*n* > 4, *p* <  0.05) compared to PBS group

To investigate the mechanisms of increased liver metastasis by EGb, cell mitosis and proliferation within tumor metastases were examined. Number of mitotic figures (cells undergoing mitosis within a given field) was evaluated under a light microscope with 20× magnification for H&E-stained tissue sections. Overall, there were significantly more mitotic figures in tumors from the EGb group than PBS group when numbers from all tissues were combined (Fig. 2a, *p* < 0.05). Specifically, EGb significantly increased the number of mitotic figures in splenic tumors (black bars, Fig. 2a, *p* < 0.05) but not in tumors in either liver or other tissues (gray-shaded bars for liver and open bars for other tissues, Fig. 2a). Effects on mitosis by EGb treatment were further investigated using an antibody against phospho-histone H3 Serine 10 (H3S10p), another mitotic marker in metastatic liver tumors. H3S10p was located in proliferating tumor cells that surround the necrotic areas (Fig. 2b), and EGb significantly increased H3S10p in the metastatic liver compared to PBS treatment (Fig. 2c, *p* < 0.05).

Flow Cytometry was performed to examine cell cycle progression (G1, S, and G2/M) in liver of PBS and EGb-treated mice with tumors (Fig. 2d). EGb significantly decreased the percentage of cells in G1 phase when compared to PBS control. It had no effect on the percentage of cells in G2/M phase in liver with tumors (Fig. 2e, *p* < 0.05).

Ki67, a proliferation marker, was analyzed to determine the growth of tumor in the metastatic liver. Ki67-positive cells were primarily located in the proliferating areas of metastatic tumor surrounding the necrotic areas (Fig. 2f). Moreover, EGb significantly increased the percentage of Ki67-positive cells in liver tumors when compared to tumors from PBS controls (Fig. 2g, *p* < 0.05). Overall, EGb significantly increased proliferation of metastatic tumor cells in mouse liver when compared to PBS treatment.

### EGb altered mRNA expression of cell cycle, metastasis, apoptosis, oxidative stress and mitochondrial function related genes in mouse liver

**Fig. 3:**
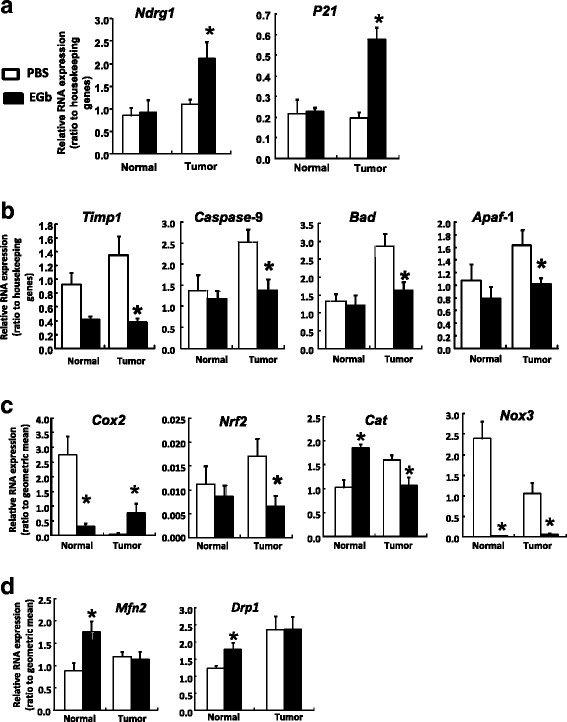
Relative mRNA expression in mouse liver. mRNA expression was analyzed by two-step real-time RT-PCR using primers that are specific to mouse genes. All data were normalized to geometric mean of housekeeping genes including L7a, Tbp, 36B4, Actb, HPRT and GAPDH. BLI imaging at the beginning of EGb treatments was used to determine tumor development and to group animals into Tumor (animals with tumor development) and Normal (animals without detectable tumors). Open bars represent the PBS treated control group, and filled bars represent EGb treated group. **a** Expression of cell cycle and metastasis related genes in mouse livers. **b** Expression of apoptosis related genes. **c** Expression of oxidative stress related genes. **d** Expression of mitochondria function related genes. Data represent means ± SEM, *n* > 5. Asterisks (*) indicate statistical significance (*p* < 0.05) when compared to the PBS group

In order to determine the potential mechanism of the increased metastasis from the primary splenic site to the liver by EGb, liver tissues were collected from both Tumor and Normal groups to investigate various pathways that could affect hepatic microenvironment and impact tumor physiology. EGb-treated mice had significantly increased mRNA expression of *Ndrg1* and *p21*, two metastasis-related genes, when compared to the PBS control, but only in animals with liver tumors (Tumor), and not in the Normal, non-tumor groups (Fig. 3a, *p* < 0.05).

mRNA expression of apoptosis-related genes *Timp1*, *Caspase-9*, *Bad* and *Apaf1* were also assessed*.* EGb-treated mice had significantly decreased mRNA expression of these genes when comparing to PBS controls but only in animals with liver tumors, and not in the Normal group (Fig. 3b, *p* < 0.05).

EGb treatment exerted differential patterns of mRNA expression of oxidative stress-related genes. Specifically, EGb significantly decreased gene expression of *Nrf2*, *Cat* and *Nox3*, in the liver of mice with tumors (Tumor, Fig. 3c, *p* < 0.05). On the other hand, EGb significantly increased *Cox2* mRNA expression in the Tumor group when compared to the PBS control group. Interestingly, in the Normal group, EGb significantly decreased mRNA expression of *Cox2* and *Nox3* while Cat expression was upregulated in the liver of EGb-treated mice without tumors (Normal, Fig. 3c).

EGb significantly increased mRNA expression of genes related to mitochondrial functions including Mitofusin-2 (*Mfn2)* and dynamin related protein-1 (*Drp1)* in Normal group when compared to the PBS control group (*p* < 0.05), but there were no significant changes observed in the Tumor group.

### EGb activated MAPK signaling pathways in mouse metastatic liver tumors

**Fig. 4:**
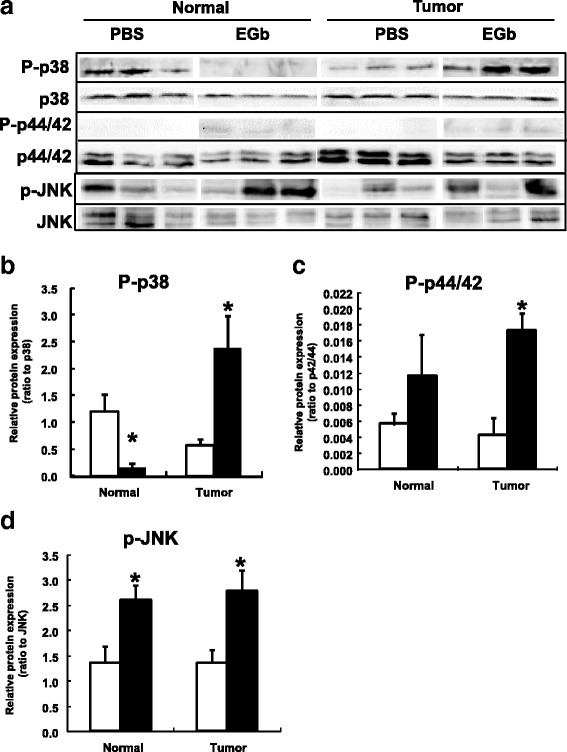
Western blot analysis of proteins in the mitogen-activated protein kinase (MAPK) pathway in mouse liver. **a** Representative western blots of total and phosphorylated MAPK family proteins p38, p44/42, and JNK. Normal represents liver samples of animals without any tumor, while Tumor represents liver samples of animals with tumors. **b**, **c**, **d** Quantification of phosphorylated p38, p44/42, and JNK, respectively. Open bars represent the PBS control group, and filled bars represent EGb-treated group. Y-axis represents the ratio of the phosphorylated protein to the total level of respective protein. Values are means ± SEM, *n* > 5. Asterisks (*) indicate statistical significance (*p* < 0.05) when compared to the PBS group

Western blot was performed to investigate the activation of the MAPK/JNK signaling pathway, a major signaling pathway in tumor development in mouse liver with metastasis (Fig. 4a). MAP kinases in this pathway include extracellular signal-regulated kinases, c-Jun N-terminal or stress-activated protein kinases (JNK), ERK/big MAP kinase 1 (p44/42), and the p38 group of protein kinases (p38). EGb significantly increased hepatic protein level of phosphorylated p38 (p-p38), phosphorylated p44/42 (p-p42/44) and phosphorylated JNK (p-JNK) in the Tumor group when compared to the PBS control (Fig. 4b–*p* < 0.05). In the Normal group, EGb ablated p-p38 and increased protein level of p-JNK (Fig. 4b, d). In conclusion, EGb activated MAP kinase cascades in the Tumor group, and exerted different effects in mice without tumor development (Normal group).

## Discussion

The present study aimed at investigating effects of Extract of *Ginkgo biloba* (EGb) on liver metastasis of colon cancer in a mouse xenograft model. Our results demonstrated that EGb exaggerated metastasis to liver from the spleen. The increased metastasis was accompanied by a decreased necrosis of tumor and apoptosis of tumor cells in mouse liver. Moreover, EGb increased mitosis and promoted cell cycle progression in liver, leading to the increased proliferation of tumor cells in mouse. Our results suggested that EGb increased metastasis by decreasing apoptosis and necrosis, increasing mitosis and proliferation of tumor cells. EGb altered expression of key genes in the oxidative stress pathway and activated the MAPK cascades, a stress-related pathway in mouse liver.

A commercially produced Ginkgo extract, EGb761, showed conflicting results in a clinical trial in which increased risks of breast and colon cancers and reduced risk of prostate cancer were reported [32]. Also, EGb increased incidence of hepatocellular adenomas and carcinomas in rodents [36, 37]. The increased risks of cancers by EGb could be explained by bioactive compounds in the extract. Quercetin, one of the major bioactive components, significantly increased tumor incidence in renal tubule in male rats [38]. Harwood et al. summarized the studies of carcinogenicity of quercetin in rats, mice and hamsters [39]. Hard et al. further showed that quercetin exerted carcinogenicity in chronic progressive nephropathy patients [40]. In our study, the EGb contained higher amount of quercetin, with 57.2% of total flavonol glycosides compared to EGb761 (47.3%). This supports the previous studies related to the carcinogenicity of quercetin and potentially explains the effects on exaggerated liver metastasis by EGb in the present study.

Necropsy and pathological results from liver demonstrated that EGb promoted liver metastasis in this mouse model of colon cancer metastasis. EGb significantly increased numbers of metastatic tumors in liver. This could be explained by the effects of EGb on increased tumor cell mitosis and proliferation, as well as decreased early apoptosis [15, 17].

It has been shown that EGb altered expression of genes in *Nrf2*-mediated oxidative stress response pathway, *Myc* gene-centered network in mice [41], and Wnt signaling pathway associated genes in hepatocellular carcinoma [36]. This is confirmed by the EGb-induced reduction in mRNA expression of *TIMP1*, *Caspase9*, *Bad*, *Apaf1*, *Nrf2*, *Cat* and *Nox3* in the Tumor group. N-myc downstream-regulated gene 1 ( *Ndrg1* ) was related to tumor progression and metastasis [42, 43] and its induction by EGb in the present study is potentially the key factor that interacts with both WNT pathway and Myc network. In addition, EGb increased mRNA expression of Cox2 in mice with liver tumors. Since Cox2 is involved in liver disease and hepatic tumorigenesis [44–46], induction of Cox2 by EGb in liver with tumors in the current study could be an important indicator of tumor-promoting effects from EGb. Together with the contradictory data from mice without tumors (Normal group), these confirmed the previous published data showing anti-oxidative and anti-apoptotic effects of EGb. However, these beneficial effects observed in normal aging animals may also benefit tumor growth in the current study.

In the present study, mRNA expression of mitochondrial function related genes, *Mfn2* and *Drp1* were increased in the Normal group, but not in the Tumor group, suggesting that EGb may enhance mitochondrial function in tissues under normal conditions, but not in the Tumor group during or after the metastasis. Therefore, in liver metastasis, especially in tumor bearing liver, EGb has lost its beneficial effects in enhancing mitochondrial functions.

Through the investigation, it is apparent that various MAPK cascades have been activated in the EGb-treated liver with tumors. EGb activated ERK kinase pathway in the metastatic mouse liver, confirming the cell cycle promoting effects that were observed in the EGb group [47]. JNK pathway, another branch of MAPK pathway, was also activated, possibly through the activation of its regulator *Ndrg1* [43]. Increased phosphorylation of p38 stabilized *p21* in vivo [48], correlated well with the increased *p21* by EGb observed in the Tumor group in the present study. On the contrary, in normal liver without tumor, phonsphorylation of p38 protein was inhibited by EGb treatment, confirming the reported inhibitory effect from EGb in normal liver which offers protection against liver fibrosis [49]. Correlation between EGb-induced phospho-JNK, phospho-p38, and the downregulation of apoptosis-related genes *Caspase9* and *Bad* was also well documented in the current study [50]. The coordinated increase of phosphorylation of ERK1/2 (p44/42), p38, and JNK1/2 and the increased *Cox2* by EGb in the liver with tumors further illustrated the interactions between the MAPK signaling and oxidative stress [51]. These further validated the hypothesis that in liver with metastatic tumors, the EGb treatment has lost its beneficial effects as reported previously in normal liver.

## Conclusions

EGb promoted tumor cell metastasis by enhancing cell cycle progression and cell proliferation, and inhibiting early apoptosis, resulting in the development of liver metastasis of colon cancer in a mouse model. This may potentially be associated with the increased MAPK signaling pathway in mouse liver. Classifying these bioactive compound-sensing mechanisms in liver may help to monitor drug interactions in cancer patients in response to the consumption of EGb. Future studies are needed to further illustrate the timing of the effects from EGb treatment on various signaling pathways in liver in order to better understand the health benefits from EGb and to avoid the adverse effects on cancer metastasis.

## Additional file

Additional file 1: Table S1. List of primers for PCR. **Table S2** List of antibodies. (DOCX 17 kb)

## Abbreviations

BLI: Bioluminescent imaging
EGb: Extract of *Ginkgo biloba*
H&E: Hemotoxylin and eosin
H3S10p: Phospho-histone H3 serine 10
p-JNK: Phospho-c-Jun N-terminal kinase
p-p38: Phospho-p38
p-p42/44: Phospho-p42/44
