# Supplementary material for: Extract of Ginkgo biloba exacerbates liver metastasis in a mouse colon cancer Xenograft model
Source: BMC Complement Altern Med. 2017 Dec 2;17:516. doi: 10.1186/s12906-017-2014-7 (PMC5712166; doi:10.1186/s12906-017-2014-7)
Supplement: Additional file 1: Table S1. — List of primers for PCR. Table S2 List of antibodies. (DOCX 17 kb) [file 12906_2017_2014_MOESM1_ESM.docx]

| Gene (Enzemble No.Transcript ID) | **mRNA Sequence** | |
| --- | --- | --- |
| mNDRG1 (ENSMUST00000168979) | Forward +238F | CTC ACG TAT CAC GAC ATC GGC A |
|  | Reverse +310R | CCT GCA TGT CCT CAG AGT TGA AGA G |
| mP21 (ENSMUST00000023829) | Forward +32F | CAG AGT CTA GGG GAA TTG GAG TCA G |
|  | Reverse +97R | CCT GTG GCT CTG AAT GTC TGG A |
| mTIMP1 (ENSMUST00000115342) | Forward +391F | TGG GAA ATG CCG CAG ATA TCC |
|  | Reverse +463R | GAC TTG TGG GCA TAT CCA CAG AGG |
| mCaspase-9 | Forward F | TGT CTT TAA AGC GAT TCT GC |
|  | Reverse R | AGT GGA CTT TCA CAA AGC AA |
| mBad | Forward F | CAG CCC AGA GTA TGT TCC AG |
|  | Reverse R | AGG CCC CTA TCT GTA GCA CT |
| mApaf-1 | Forward F | CTC TAG ATG AAG CCA TGT CG |
|  | Reverse R | AAG GAT GGA AAG GTC TGT GT |
| mCox2 (ENSMUSE00000262815) | Forward +943F | GCC TGG TCT GAT GAT GTA |
|  | Reverse +1019R | GAT GCT CCT GCT TGA GTA |
| mNrf2 (ENSMUSE00000165597) | Forward +634F | AGT CGC TTG CCC TGG ATA |
|  | Reverse +716R | AGA GCT ATT GAG GGA CTG GG |
| mCat (ENSMUSE00000228063) | Forward +325F | GGA TTC CTG AGA GAG TGG TA |
|  | Reverse +413R | TGC CTT GGA GTA TCT GGT |
| mNox3 (ENSMUSE00000135410) | Forward +426F | CAA CCT GGA GCG TTA TCA CC |
|  | Reverse +542R | TCA AAG GTG CGG ACT GGA |
| mMfn2 (ENSMUSE00001267859) | Forward +1177F | GAT CTT CTT CGT GTC TGC CAA GGA |
|  | Reverse +1273R | TTG AAA ACC TTC TGC GAG AGC G |
| mDrp1 (ENSMUSE00000436538) | Forward +141F | TTG GGG GTC CAG TGT TTC GG |
|  | Reverse +228R | GTC CGC ACC CAC TGT GTT GAA G |
| mRpl7a (ENSMUST00000102898) | Forward +176F | AAC TTC GGC ATT GGA CAG GAC A |
|  | Reverse + 289R | TTT GAG CCG CTT GTA GAG GAT AGC |
| mGAPDH ( ENSMUSE00001143324) | Forward +149F | CCA GCT TGT TCC TTC AGA CC |
|  | Reverse +234R | GCC CTC AAG GAC AAA GAC AG |
| mTbp (ENSMUST00000162505) | Forward +204F | AGG ATG CTC TAG GGA AGA TCT GAG |
|  | Reverse +281R | GAG CAT AAG GTG GAA GGC TGT T |
| mActb (ENSMUSE00001272130) | Forward +247F | AAT GGG TCA GAA GGA CTC CT |
|  | Reverse +312R | TTC AGG GTC AGG ATA CCT CT |
| m36b4 (ENSMUST00000086519) | Forward +502F | CAC TGG TCT AGG ACC CGA GAA G |
|  | Reverse +523R | GTG ACC AGA TCC TGG GCT CTT C |
| mHPRT ( ENSMUSE00000506191) | Forward +111F | CGT GAT TAG CGA TGA TGA AC |
|  | Reverse +178R | CCT CGG CAT AAT GAT TAG GT |

**Additional file 1: Table S1 List of primers for PCR**

**Additional file 2: Table S2 List of antibodies**

| Name | Cat. Number | Manufacturer |
| --- | --- | --- |
| Phospho-p38 MAPK(Thr180/Tyr182)(D3F9) Rabbit mAb | 9910 | Cell Signaling Technology, Inc. |
| p38 MAP Kinase Antibody | 9926 | Cell Signaling Technology, Inc. |
| Phospho-p44/42 MAPK(Thr202/Tyr204)(D13.14.4E) Rabbit mAb | 9910 | Cell Signaling Technology, Inc. |
| p44/42 MAP Kinase(137F5) Rabbit mAb | 9926 | Cell Signaling Technology, Inc. |
| Phospho-SAPK/JNK(Thr183/Tyr185)(81E11) Rabbit mAb | 9910 | Cell Signaling Technology, Inc. |
| SAPK/JNK(56G8) Rabbit mAb | 9926 | Cell Signaling Technology, Inc. |
| Actin(I-19)-R | sc-1616-R | Santa Cruz biotechnology, Inc, |
| Ki-67 | 550609 | BD Biosciences |
| p-Histone H3(Ser10) | sc-8656-R | Santa Cruz biotechnology, Inc. |
